# Extra-Uterine Pregnancy

**Published:** 1890-01

**Authors:** E. McD. Bridgford

**Affiliations:** 1751 N. 11th street, St. Louis, Mo.


### EXTRA-UTERINE PREGNANCY.

ST. LOUIS, MO., Dec. 18, 1889.

*Editor Daniel's Texas Medical Journal:*

"Will wonders never cease?" is the question every physician who has read or heard of Dr. Lowry's remarkable case of extra-uterine pregnancy, is asking. The case is certainly one of great interest to the medical fraternity, and I do hope it will be thoroughly discussed through your valuable journal. I have not had the pleasure of reading Dr. Lowry's report of the case reviewed by Dr. Cross, so I will have to content myself with a few words on some points brought out by the latter gentleman.

I think Dr. Lowry is to be congratulated that the introduction of *a large blunt sound* was not followed by unpleasant symptoms. The passage of such an instrument into the uterine cavity is not always an easy task, and to pass the sound through the *Fallopian tube*, is to my mind impossible. Now, if Dr. Lowry will kindly inform us, how he managed to get the sound into the uterus *five inches* and then *nine inches* through the *Fallopian tube* —altogether *fourteen inches*—or, about the usual length of a uterine sound, I think it would be quiet interesting to your many readers. Dr. Thomas, in "Thomas on the Diseases of Women,"

page 772, records seventeen cases of extra-uterine pregnancy. The fœtus, in one case, was discharged per vagina. The author failed to state the duration of pregnancy, but says the patient recovered. Hoping we may hear more of this interesting case, and wishing Editor Daniel and the readers of the JOURNAL "a merry Christmas and happy New Year,"

I am fraternally yours,

E. McD. BRIDGFORD, M. D.,

1751 N. 11th street,

St. Louis, Mo.

